# Characterizing extravascular lung water—A dual-contrast agent extracellular volume approach by cardiovascular magnetic resonance

**DOI:** 10.1016/j.jocmr.2025.101883

**Published:** 2025-03-20

**Authors:** Felicia Seemann, Rim N. Halaby, Andrea Jaimes, Kendall O’Brien, Peter Kellman, Daniel A. Herzka, Robert J. Lederman, Adrienne E. Campbell-Washburn

**Affiliations:** a Cardiovascular Branch, Division of Intramural Research, National Heart, Lung, and Blood Institute, National Institutes of Health, Bethesda, Maryland 20892, USA; b Department of Radiology, Case Western Reserve School of Medicine, Cleveland, Ohio 44106, USA

**Keywords:** Lung water, Ferumoxytol, Extracellular volume, Dual-agent

## Abstract

**Background:**

Pathological extravascular lung water is a facet of decompensated congestive heart failure that current cardiovascular magnetic resonance (CMR) methods fail to quantify. CMR can measure total lung water density, but cannot distinguish between intravascular and extravascular fluid, and thus is not diagnostic. Therefore, we develop and evaluate a novel method to measure extravascular lung water by distinguishing intravascular from extracellular fluid compartments using two different contrast agents, extracellular gadolinium chelates and iron-based intravascular ferumoxytol.

**Methods:**

We created two porcine models of pulmonary edema: reversible catheter-induced mitral regurgitation to induce extravascular lung water (n = 5); intravascular volume overload using rapid colloid infusion (n = 5); and compared to normal controls (n = 8). We sequentially acquired lung T1 maps and lung water density maps at 0.55T with native, gadolinium-based, and ferumoxytol contrast, from which we calculated the extracellular volume fraction (ECV) and blood plasma volume fraction in the pulmonary tissue, respectively. We computed extravascular ECV as the difference in ECV and plasma volume fractions. Extravascular lung water volumes were estimated.

**Results:**

In the mitral regurgitation model, baseline vs mitral regurgitation ECV_extravascular_ increased from 27 ± 4.1% to 32 ± 1.9% (p = 0.006), and extravascular lung water volume increased from 105 ± 19 mL to 143 ± 15 mL (p = 0.048). Plasma volume fraction was similar at baseline vs mitral regurgitation (43 ± 4.2% vs 46 ± 5.4%, p = 0.26). Compared to naïve pigs, we measured higher plasma volume fractions in the intravascular volume-loaded model (42 ± 4.7% vs 51 ± 2.7%, p = 0.0054), but no differences in ECV_extravascular_ (21 ± 4.6% vs 21 ± 3.6%, p = 0.99) or extravascular lung water volume (67 ± 13 mL vs 89 ± 24 mL, p = 0.11). Assessing the regional distribution, the plasma volume was higher posteriorly, indicating gravitational dependency, whereas, the extravascular lung water was higher anteriorly.

**Conclusion:**

Extravascular lung ECV measurements and derived lung water volumes corresponded well with predicted increases in extravascular and intravascular pulmonary fluid in animal models. This method may enable mechanistic studies of lung water in patients with dyspnea.

## Graphical abstract

**Figure:**
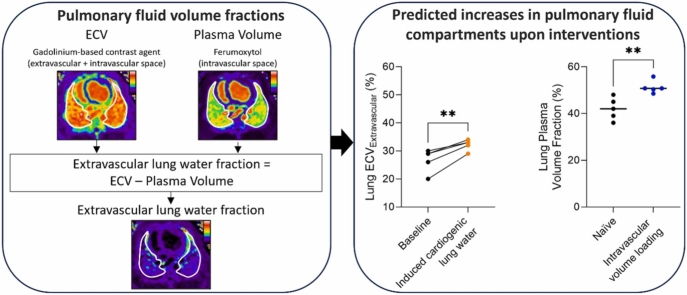

## 1 Introduction

The human body is composed of ∼50–60% water by mass which can be partitioned into two main compartments of intracellular and extracellular fluid [1]. The extracellular fluid, i.e. fluid outside the cells, can be further divided into the intravascular (blood plasma) and extravascular (interstitial) compartments. Dyspnea caused by cardiogenic pulmonary edema, or lung water, a hallmark of decompensated congestive heart failure, is attributed to an extracellular fluid overload in the extravascular pulmonary interstitium [2].

Proton density-weighted cardiovascular magnetic resonance (CMR) can quantify lung water in three-dimensional (3D), providing means to monitor and predict outcomes in heart failure [3], [4], [5], [6], [7], [8], [9], [10]. However, proton density-weighted CMR cannot distinguish between normal intravascular fluid, i.e. blood, and the extravascular pulmonary interstitial fluid which is specifically associated with pulmonary edema [3], [4], [5], [8]. Extravascular lung water can be assessed using semi-quantitative ultrasound B-lines or invasive thermodilution, none of which provide 3D resolution [11]. Nuclear medicine imaging can differentiate between intra- and extravascular lung water by subtracting the activity from two tracers; one that equilibrates in both the intravascular and extravascular space vs another in only the intravascular space [12]. But nuclear imaging provides limited spatial resolution and exposes patients to ionizing radiation.

Extracellular volume fraction (ECV) mapping by CMR is a well-established noninvasive, quantitative, ionizing radiation-free technique to measure the myocardial extracellular fractional distribution volume [13], [14], [15], [16], [17]. ECV maps are derived using cardiac T1 maps acquired before and 10–30 min after injecting a gadolinium-based contrast agent, a chelate that accumulates in the entire extracellular space [17], [18]. CMR myocardial ECV mapping is sensitive to both intravascular and extravascular changes in extracellular volume, and is typically used to assess extracellular matrix expansion in the tissue such as by amyloidosis or fibrosis [15], [19], [20]. Another contrast agent used in CMR is ferumoxytol, an iron-oxide nanoparticle that remains purely intravascular for hours after infusion, with a 9–21 h intravascular half-life [21], [22]. Ferumoxytol is used off-label for angiography [23], [24], blood volume quantification [25], and four-dimensional flow imaging [26], [27]. We hypothesize that the extravascular ECV component can be estimated from the differences between gadolinium-based ECV and ferumoxytol-based blood plasma volume fractions.

Hence, we utilize the differential compartmentalization of gadolinium-based and ferumoxytol contrast agents to develop a novel CMR method that isolates and quantifies extravascular interstitial lung water using an ECV approach. We test our hypothesis by applying this method in porcine models where we experimentally induce increases in extravascular and intravascular pulmonary fluid.

## 2 Materials and methods

### 2.1 Animal models

Experiments were approved by the Institutional Animal Care and Use Committee and conducted per contemporary guidelines from the National Institutes of Health. We performed 18 porcine experiments in female juvenile pigs, maintained under general anesthesia with mechanical ventilation and isoflurane inhalation. Percutaneous intravascular access was placed in the femoral artery, femoral vein, and ear vein. A fluid-filled 7F Swan-Ganz catheter was placed in a main pulmonary artery branch through femoral venous access and used to measure pulmonary arterial wedge pressure (PAWP) using an air-filled balloon. We then acquired CMR images with native, gadolinium-based, and ferumoxytol contrast in two animal models described below: increased extravascular lung water (n = 5), increased intravascular volume loading (n = 5), and naïve controls (n = 8).

#### 2.1.1 Extravascular lung water model

We increased extravascular lung water using a previously published model of acute severe mitral regurgitation, created and controlled by applying tension on an externalized suture placed across the anterior mitral leaflet without surgery under X-ray guidance, in a procedure resembling LAMPOON mitral leaflet laceration [8], [28]. A regurgitant jet was dynamically induced inside the CMR scanner by applying tension on the suture, triggering accumulation of extravascular lung water as a result of an increased left atrial and pulmonary vein blood pressure [29]. The model is reversible, allowing induction and relief of mitral regurgitation, as well as repeated exposures. This enables imaging at baseline and regurgitation after each contrast administration, meaning that these pigs serve as their own controls. The reproducibility of the repeated inductions of mitral regurgitation was assessed by comparing the mitral regurgitant fractions by CMR and change in PAWP between baseline and the valvular openings.

#### 2.1.2 Intravascular volume loading model

We created a model of increased intravascular volume by infusing 15 mL/kg of hydroxyethyl starch (Hespan, 6% Hetastarch in 0.9% Sodium Chloride, Hospira Inc, Lake Forest, Illinois), a colloid fluid with osmotic propensity to remain in the intravascular space [30], [31], [32]. As this method of volume loading could not be reversed between contrast administrations, naïve animals were used as controls during analysis.

### 2.2 Experimental imaging protocol

**Fig. 1:**
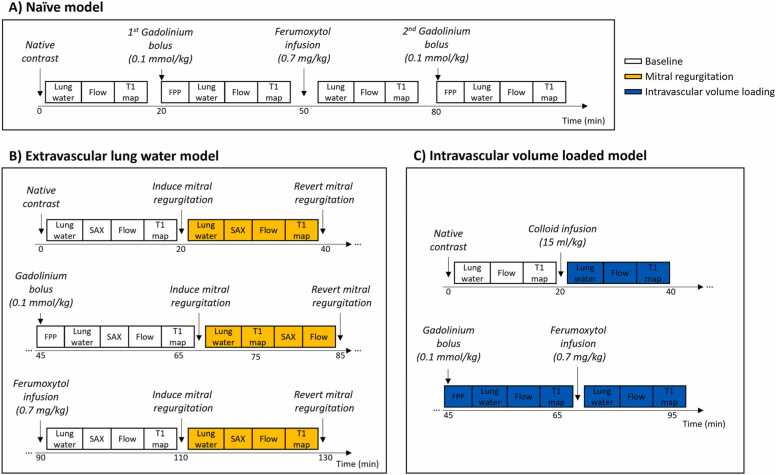
The experimental protocol consisted of sequential imaging phases with native, gadolinium-based, and ferumoxytol contrast. Each imaging phase consisted of equal blocks in which lung water, flow, and T1 maps, were acquired. A first-pass perfusion (FPP) image was acquired during the injection of the gadolinium-based contrast agent bolus to calculate pulmonary blood volume. Short-axis cine (SAX) images were acquired in the mitral regurgitation model, for computation of the mitral regurgitant fraction. (A) Naïve animal protocol, consisting of four imaging blocks. The first imaging block with gadolinium-based contrast agent administration was omitted in n = 3 animals. (B) Protocol of the extravascular lung water model, induced through reversible mitral regurgitation. (C) Intravascular volume-loaded protocol, induced through colloid infusion.

The overall experimental protocol consisted of three sequential imaging phases with native, gadolinium-based (Gadobutrol, Gadavist, Bayer Healthcare, Whippany, New Jersey), 1.81 h terminal half-life, 0.1 mmol/kg, bolus flushed in 20 mL saline at 4 mL/s through ear vein access), and ferumoxytol (Covis Pharma, Waltham, Massachusetts) (0.7 mg/kg diluted in 50 mL saline, infused into the femoral access at ∼0.4 mL/s) contrast as illustrated in Fig. 1. Ferumoxytol was administered ∼30 min following gadolinium-based contrast agent administration. To explore the impact of the order in administering contrasts, a second gadolinium-chelate bolus was administered ∼30 min after infusing ferumoxytol in n = 5 naïve animals. In the remaining n = 3 naïve pigs, we omitted the first gadolinium-chelate bolus, i.e. first administered ferumoxytol then gadolinium-based contrast agents.

Each imaging phase included lung water density maps, T1 maps, and flow images. Typical pulse sequence parameters are listed in Table 1. We imaged the main pulmonary artery flow in naïve animals and aortic flow in the other models, to enable quantification of the mitral regurgitation. A first-pass perfusion image was acquired during administration of the gadolinium-chelate bolus to quantify pulmonary blood volume. T1 maps after ferumoxytol administration were acquired at least 1 h after the gadolinium-based contrast bolus, i.e. double the recommended upper limit time (30 min) for ECV quantification [15], [33], to minimize the residual T1 shortening from gadolinium chelates. A short-axis cine stack covering the ventricles was acquired within each imaging block in the model with mitral regurgitation, for calculation of the mitral regurgitant fraction. PAWP was measured in the extravascular and volume-loaded models to corroborate increases in pulmonary fluid. Femoral arterial hematocrit was measured in direct conjunction with the CMR protocol.

**Table 1:** Typical pulse sequence parameters for the acquired images.

	SASHA	Lung water	Flow	Short-axis cine	First-pass perfusion
TE (ms)	1.18	0.56	4.44	1.35	0.78
TR (ms)	3.0	9.0	7.14	3.3	2.5
Flip angle (°)	80	1	30	80	31
Field of view (mm)	302 × 393	450 × 450 × 252	330 × 310	360 × 270	450 × 450 × 160
Spatial resolution (mm)	3.5 × 3.5 × 10	3.5 × 3.5 × 3.5	1.7 × 1.7 × 8.0	1.9 × 1.9 × 8.0	3.5 × 3.5 × 20
Acquisition time (min)	0.35	2.5	2	5	1

*Data are the pulse sequence parameters used in this study, SASHA* saturation recovery single-shot acquisition, *TE* echo time, *TR* repetition time

Imaging was performed on a 0.55T CMR system (prototype MAGNETOM Aera, ramped down from 1.5T, Siemens Healthineers, Erlangen, Germany) with an 18-channel spine-phased array receiver coils retuned for 0.55T and a prototype 12-element chest coil specifically designed for 0.55T cardiac and lung imaging (NeoCoil, Pewaukee, Wisconsin) [34], [35].

T1 maps were acquired in three axial slices depicting the lungs and left ventricular blood pool, using an electrocardiogram (ECG)-gated saturation recovery single-shot acquisition (SASHA) balanced steady-state free precession (bSSFP) sequence at end expiration with saturation times 104 ms × 8 averages, 200 ms × 4 averages, 374 ms × 4 averages, and 43 segments [36]. We chose SASHA due to its reduced sensitivity to off-resonance and high accuracy [37]. Acquiring T1 maps at end-expiratory breath-holds minimized the impact of respiratory phase variation on lung T1 [38]. Bloch equation and Monte Carlo simulations were performed to demonstrate the sensitivity to a range of physiological T1 values pre and post contrast at 0.55T (Supplemental Material).

We measured lung water from coronal images using a free-breathing three-dimensional (3D) proton density-weighted ultrashort echo time gradient echo stack-of-spirals sequence with 171 spiral interleaves, 5 ms readout duration, and superior-inferior respiratory navigation readouts every 200 ms [39]. Lung water images were reconstructed to the end-expiratory respiratory phase using a motion-compensated image reconstruction [8].

Cross-sectional main pulmonary artery and aortic flow were measured using free-breathing through-plane phase contrast gradient echo with retrospective ECG-gating (velocity encoded 150 cm/s, bandwidth 299 Hz/Px). Left ventricular volumes were measured from a short-axis cine stack covering the ventricles, acquired at free-breathing using a retrospectively ECG-gated free-breathing binned bSSFP sequence [40].

The first pass of a 2 mL gadolinium-chelate bolus through the lungs was captured at end expiration by dynamic contrast-enhanced imaging in the coronal view, using a 3D Cartesian gradient echo sequence at end expiration with frame rate 1.5 s/volume. A second bolus was injected immediately after this acquisition, to achieve a total gadolinium-based contrast agent dose of 0.1 mmol/kg.

### 2.3 Image analysis

We performed image analysis using the software Segment v3.1R8154 (Medviso, Lund, Sweden) [41]. We used automated tools to derive time-resolved delineations of the left ventricular endocardium and vascular cross section in cine and flow images, respectively, and subsequently calculated stroke volume, cardiac output, and mitral regurgitant fraction $\left(\frac{{\mathrm{SV}}_{\mathrm{left ventricle}}-{\mathrm{SV}}_{\mathrm{aorta}}}{{\mathrm{SV}}_{\mathrm{left ventricle}}}\right)$. Manual corrections of contours were performed as necessary.

Whole lung water density maps, excluding major vasculature, were calculated using a previously described automated image processing pipeline [5], [8]. Briefly, the lung segmentation is performed using a trained neural network with a U-net architecture, followed by a signal-intensity-based coil shading correction and pixel-wise lung water density calculation as the ratio of pulmonary signal intensities to the median signal intensities in the surrounding body tissues. Lung volume across both lungs was computed as the volume encompassed within the lung segmentations. Global lung water density was defined as the average lung water density across both lungs, and total lung water volume was computed as

Lung water volume=Lung water density∙Lung volume.

We manually placed a region of interest in the left ventricular blood pool in the T1 maps, avoiding partial volume effects and papillary muscles, and computed the average blood T1. The average lung T1 was calculated as the mean T1 within manually contoured lungs across all three slices.

Using the first-pass perfusion images, the pulmonary transit time was derived from manually placed regions of interest in the right and left ventricles [42]. We calculated pulmonary blood volume as

Pulmonary blood volume=Pulmonary transit time∙Cardiac output

Pulmonary blood volumes by first-pass perfusion were compared to the derived lung water volumes by proton density-weighted imaging.

### 2.4 Extravascular ECV

**Fig. 2:**
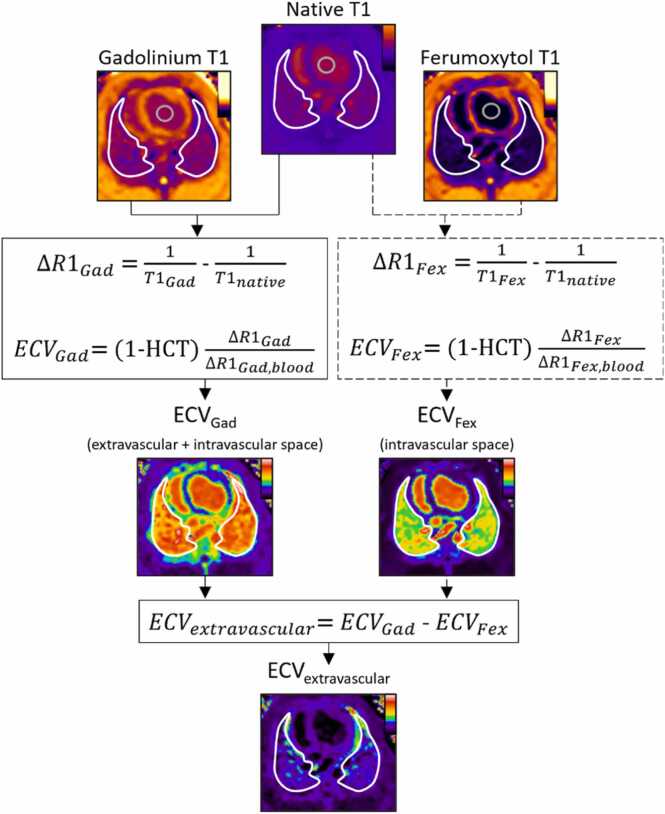
Illustration of the lung extracellular volume (ECV) and plasma volume fraction calculations using SASHA T1 maps with native, gadolinium-based (“Gad”), and ferumoxytol (“Fex”) contrast to isolate the extravascular ECV component. Regions of interest highlighting the lungs and the left ventricular blood pool are marked in white and gray, respectively. *HCT* hematocrit, *SASHA* saturation recovery single-shot acquisition, *ECV* extracellular volume fraction

We calculated lung ECV, i.e. the percentage of pulmonary tissue volume comprised of extracellular space, by measuring the change in T1 before and after contrast administration as

$$\mathrm{ECV}=(1-\mathrm{hematocrit})\frac{{∆R1}_{\mathrm{lung}}}{{∆R1}_{\mathrm{blood}}},$$

where ΔR1 = 1/T1_contrast_ – 1/T1_native_. The extravascular ECV component was derived in three steps, as illustrated in Fig. 2. First, we calculated the gadolinium-based lung ECV, which reflects both the extravascular and intravascular space in which gadolinium chelates accumulate (plasma and tissue extracellular matrix). Second, we calculated the ferumoxytol-based ECV. We refer to the ferumoxytol-based ECV as the blood plasma volume fraction, as this quantification only reflects the intravascular ECV tissue compartment (i.e. plasma). Third, we isolate the extravascular ECV component, ECV_extravascular,_ through the subtraction.

ECV_extravascular_ = ECV – Plasma volume fraction

The mathematical derivation of this equation is presented in the Supplemental Material.

We computed ECV and plasma volume fractions using region of interest-based mean T1, providing an average value across both lungs. We did not derive pixel-wise ECV or plasma volume fraction maps, as animals were repositioned for ferumoxytol infusion, resulting in slight changes to imaging planes. To ensure consistent physiology when calculating ECV and plasma volume fractions in the model with mitral regurgitation, we separately paired baseline and mitral regurgitation T1 maps (Fig. 2B). For the intravascular volume-loaded model, we used native T1 maps acquired after colloid fluid infusion but before contrast injections to calculate ECV.

Moreover, assuming the calculations in the three axial slices are representative of the whole lung ECV, we calculated the extravascular lung water volume as

Extravascular lung water volume = ECV_extravascular_∙ lung water volume

To investigate any gravitational dependence of the pulmonary fluid compartments, we divided the lungs into three equal parts in the anteroposterior direction, and separately calculated ECV, plasma volume fractions, and ECV_extravascular_ for each section.

#### 2.4.1 Interpretation of lung ECV

**Fig. 3:**
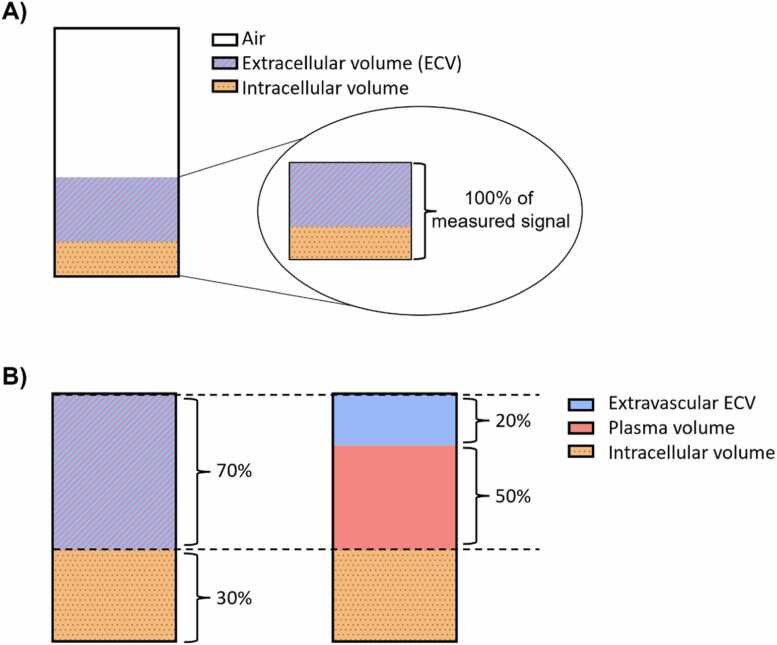
Conceptual illustration of a three-compartment model related to extracellular volume (ECV) assessments in the lungs. (A) Schematic illustration of the compartments summing up to the total volume of the lungs, including air, ECV, and intracellular volume. The quantitative volume fractions by CMR-based ECV and plasma volume mapping are insensitive to air, meaning that 100% of the measured volume fraction is ascribed to the sum of the ECV and the intracellular volume. (B) In this study, we further subdivide the ECV compartment into two additional partitions, the extravascular and intravascular (plasma) compartments. We did not study the compartmentalization of the extravascular vs intravascular intracellular volume (i.e. tissue cells vs red blood cells), but the intracellular volume fraction of blood can be estimated as the hematocrit. The stated percentages represent typical lung volume fractions measured in this study. *CMR* cardiovascular magnetic resonance

The interpretation of lung ECV differs from myocardial ECV, given that a significant pulmonary volume fraction is comprised of air, to which CMR is insensitive. This implies that quantified ECV explicitly excludes the air volume in the compartments, meaning that the ECV in a region of interest does not describe a percentage of the total delineated volume, but rather the volume fraction of all depicted tissue after subtracting the air volume. We illustrate this concept in Fig. 3. Lung water density mapping does, however, include air volume in the density quantification (lung water density = fluid content/total lung volume). In other words, signal intensity in proton density-weighted images is highest at expiration (i.e., when lung tissue is compressed and air volume is minimal), and lowest at inspiration when lung tissue is expanded with air.

### 2.5 Statistical analysis

Statistical analysis was performed using GraphPad Prism 9 (GraphPad Software, Inc., La Jolla, California). The threshold for statistical significance was p < 0.05. Continuous variables were reported as mean ± standard deviation (SD). Paired t-tests were used to compare baseline vs regurgitation, and unpaired t-tests to compare volume loaded vs controls. T1 evolution and regional ECV were compared using one-way analysis of variance (ANOVA) with repeated measures and Geisser-Greenhouse correction. For the regional analysis, naïve animals were grouped with mitral regurgitation baseline measurements to represent a group of animals without intervention.

## 3 Results

Global lung ECV_extravascular_ calculations were feasible in all experiments. There was no difference in weight across models (naïve: 46 ± 7.3 kg, model with mitral regurgitation: 49 ± 3.6 kg, volume-loaded model: 44 ± 2.6 kg, p > 0.05) or hematocrit (naïve: 23 ± 8.8%, model with mitral regurgitation: 26 ± 3.2%, volume-loaded model: 18 ± 1.9%, p > 0.05). The lower, albeit nonsignificant, hematocrit in the volume-loaded model is attributed to the colloid fluid infusion which increases plasma volume. Table 2 summarizes T1, lung water volume, pulmonary blood volume, and cardiac parameters for each model. Mitral regurgitant fraction was not computed following the gadolinium-based contrast agent administration during mitral regurgitation in two animals, due to technical issues in acquiring short-axis cine or aortic flow images.

**Table 2:** Quantitative results in each porcine model.

	Naïve model (n = 5)						
	Native			Gadolinium chelate		Ferumoxytol	

Lung T1 (ms)	931±55			559±83		287±31	
Blood T1 (ms)	1199±85			609±105		199±21	
Lung water density (%)	33±3			34±3		32±8	
Lung water volume (mL)	310±32			312±32		295±30	
Lung volume (L)	0.93±0.14			0.93±0.15		0.95±0.20	
Pulmonary blood volume (mL)	N/A			439±67		N/A	
Cardiac output (L/min)	3.5±0.5			3.5±0.5		3.7±0.5	
Stroke volume (mL)	42±5.8			42±6.1		43±5.8	
Heart rate (bpm)	83±11			84±10		87±11	

	Extravascular lung water model (n = 5)						
	Native			Gadolinium chelate		Ferumoxytol	
	*Baseline*	*Mitral regurgitation*		*Baseline*	*Mitral regurgitation*	*Baseline*	*Mitral regurgitation*

Lung T1 (ms)	940 ±39	1025±46		399±32	448±25	269±37	295±65
Blood T1 (ms)	1207±55	1217±91		421±21	486±28	188±26	208±40
Lung water density (%)	30±2.6	31±2.7		31±2.3	32±2.6	30±1.9	31±2.3
Lung water volume (mL)	392±49	419±84		418±54	438±79	395±65	409±72
Lung volume (L)	1.33±0.22	1.33±0.21		1.37±0.22	1.36±0.17	1.33±0.25	1.30±0.27
Pulmonary blood volume (mL)	N/A	N/A		502±115	N/A	N/A	N/A
Cardiac output (L/min)	4.7±0.3	3.2±0.5		4.1±0.5	3.3±1.1	4.3±0.6	3.0±0.7
Stroke volume (mL)	46±11	27±6		43±7	30±14	42±4	26±7
Heart rate (bpm)	109±38	122±26		96±8	115±14	102±13	114±18
PAWP (mmHg)	5±2	17±5		5±2	17±4	4±2	17±5

	Intravascular volume-loaded model (n=5)						
	Native			Gadolinium chelate		Ferumoxytol	
	*Baseline*		*Volume loading*	*Volume loading*		*Volume loading*	

Lung T1 (ms)	874±41		984±41	390±38		262±21	
Blood T1 (ms)	1089±36		1249±44	393±45		189±13	
Lung water density (%)	29±3.2		32±3.5	34±3.9		32±4.1	
Lung water volume (mL)	350±67		410±62	350±56		383±59	
Lung volume (L)	1.26±0.36		1.28±0.30	1.18±0.29		1.23±0.32	
Pulmonary blood volume (mL)	N/A		N/A	592±62		N/A	
Cardiac output (L/min)	3.6±0.68		5.2±1.4	5.3±1.5		5.2±1.3	
Stroke volume (mL)	43±6.8		55±10	55±10		53±9.6	
Heart rate (bpm)	85±10		95±10	95±13		97±12	
PAWP (mmHg)	5.8±0.8		9.6±1.3	N/A		N/A	

Consecutive variables are reported as mean ± standard deviation. Reported cardiac output, stroke volume, and heart rate were derived from the flow images. Note that pulmonary arterial wedge pressure (PAWP) was not measured in the naïve animals

### 3.1 T1 evolution and contrast dynamics

**Fig. 4:**
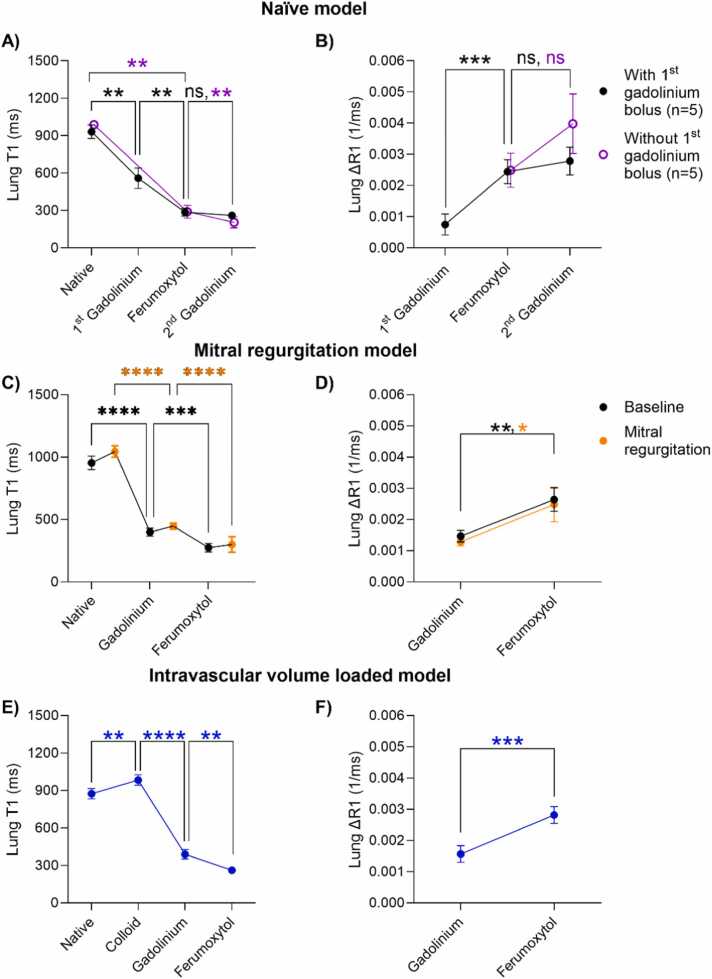
Lung T1 and ΔR1 evolution in the (A and B) naïve, (C and D) extravascular lung water induced through reversible mitral regurgitation, and (E and F) intravascular volume-loaded model, demonstrating a sequential T1 shortening and concurrent increasing ΔR1 following contrast administration. One-way ANOVA comparisons: ****p < 0.0001, ***p < 0.001, **p < 0.01, *p < 0.05, *ns* nonsignificant, *ANOVA* analysis of variance

The lung T1 and ΔR1 evolution for each model is shown in Fig. 4 and Table 2, demonstrating a sequential T1 shortening after each contrast administration (p < 0.05). Across naïve animals, we measured more lung T1 shortening between the first gadolinium-chelate bolus and ferumoxytol (ΔT1 = −272 ± 58 ms) compared to ferumoxytol and the second gadolinium-chelate bolus (ΔT1 = −26 ± 22 ms). In the n = 3 animals without the first gadolinium-chelate bolus, i.e., only ferumoxytol then gadolinium-based contrast agent administration, we also observed less T1 shortening (ΔT1 = −85 ± 6.3 ms). Native lung T1 lengthened between baseline and mitral regurgitation in the extravascular lung water model (ΔT1 = 91 ± 39 ms), as well as upon colloid fluid infusion (ΔT1 = 109 ± 34 ms). We hypothesize these T1-lengthenings are related to the increased pulmonary fluid following these interventions. Lung ΔR1, measured against native R1, differed between the first gadolinium-chelate bolus and ferumoxytol (ΔR1_1st Gadolinium_ = 0.0008 ± 0.0003 ms^−1^ vs ΔR1_Ferumoxytol_ = 0.0024 ± 0.0004 ms^−1^, p = 0.0001), but not between ferumoxytol and the second gadolinium-chelate bolus (ΔR1_Ferumoxytol_ = 0.0024 ± 0.0004 ms^−1^ vs ΔR1_2nd Gadolinium_ = 0.0028 ± 0.0005 ms^−1^, p = 0.14) nor between ferumoxytol and gadolinium chelate in the n = 3 animals without the first gadolinium-chelate bolus (ΔR1_Ferumoxytol_ = 0.0025 ± 0.0005 ms^−1^ vs ΔR1_Gadolinium_ = 0.0040 ± 0.0010 ms^−1^, p = 0.07). We therefore derived ECV_extravascular_ by administering gadolinium-based contrast before ferumoxytol and did not further analyze the n = 3 animals without the first gadolinium-chelate bolus.

### 3.2 Animal models

The mitral regurgitation intervention was reproducible, with consistent mean ± SD mitral regurgitant fractions across exam phases (native 49 ± 17%, gadolinium chelate 48 ± 18%, ferumoxytol 48 ± 21%, p > 0.05) and a consistent increase in PAWP (native ΔPAWP = 12 ± 3.2 mmHg, gadolinium-chelate ΔPAWP = 12 ± 1.9 mmHg, ferumoxytol ΔPAWP = 12 ± 3.9 mmHg, p > 0.05) for each contrast. Consistent lung water volumes at baseline imaging for each contrast indicate that enough time (∼5 min) was allowed for extravascular lung water clearance after reversing the mitral regurgitation (native 392 ± 49 mL, gadolinium chelate 418 ± 54 mL, ferumoxytol 395 ± 65 mL, p = 0.39).

In volume-loaded animals, we measured a higher pulmonary blood volume by first-pass perfusion compared to naïve pigs (592 ± 62 mL vs 439 ± 67 mL, p = 0.0057), confirming an increased intravascular volume. The volume loading was further corroborated in comparisons from baseline to after colloid infusion (Table 2), including increases in cardiac output (from 3.6 ± 0.68 L/min to 5.2 ± 1.4 L/min, p = 0.014), PAWP (from 5.8 ± 0.84 mmHg to 9.6 ± 1.3 mmHg, p = 0.009), and lung water volume (from 350 ± 67 mL to 410 ± 62 mL, p = 0.0006). There was an overall discrepancy between lung water volume and pulmonary blood volume (e.g. 312 ± 32 mL vs 439 ± 67 mL in naïve animals, p = 0.011) which might be explained by the different inherent techniques by which these are measured.

### 3.3 Dual-contrast agent ECV

Quantified ECV, plasma volume fraction, and extravascular lung water volume are summarized in Table 3. There was difference in ECV vs plasma volume fraction for the naïve animals (64 ± 1.5% vs 42 ± 4.7%, p = 0.0004), model with mitral regurgitation (baseline, 70 ± 6.7% vs 43 ± 4.2%, p = 0.0001; mitral regurgitation, 78 ± 6.9% vs 46 ± 5.4%, p < 0.0001), and volume-loaded animals (73 ± 3.1% vs 51 ± 2.7%, p = 0.0002), as expected for contrast agents with different compartment partitioning.

**Table 3:** Global pulmonary fluid quantification.

	Extravascular lung water model at baseline (n = 5)	Extravascular lung water model during mitral regurgitation (n = 5)	Naïve (n = 5)	Intravascular volume-loaded model (n = 5)
ECV (%)	70±6.7	78±6.9	64±1.5	73±3.1
Plasma volume fraction (%)	43±4.2	46±5.4	42±4.7	51±2.7
ECV_extravascular_ (%)	27±4.1	32±1.9	21±4.6	21±3.6
Extravascular lung water volume (mL)	105±19	143±15	67±13	89±24

Consecutive variables are reported as mean ± standard deviation. Note that we used the entirely naïve animals as controls for the intravascular volume-loaded model, as volume loading could not be reversed between contrast administrations

*ECV* extracellular volume

**Fig. 5:**
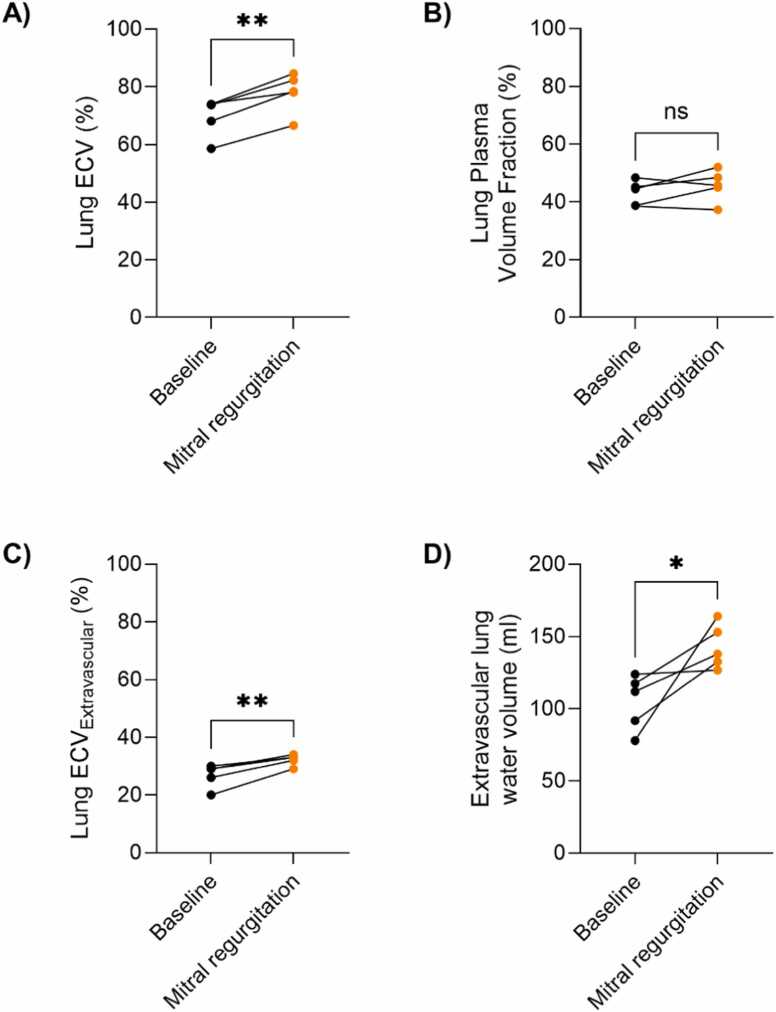
Pulmonary fluid compartments in the porcine model with mitral regurgitation. Quantified global lung (A) extracellular volume (ECV) derived using a gadolinium-based contrast agent which distributes in both the intravascular and extravascular space, and (B) plasma volume fraction using ferumoxytol, which only distributes in the intravascular space. The extravascular (C) ECV and (D) lung water volumes were higher at mitral regurgitation compared to baseline. This suggests that extravascular lung water was successfully induced and detected. Measurements at baseline (shown in black) and at mitral regurgitation (orange) were compared using a paired t-test: **p < 0.001, *p<0.01, *ns* nonsignificant

Fig. 5 shows ECV and plasma volume fraction in the model with mitral regurgitation, where an increase in extravascular lung water was expected. Between baseline and mitral regurgitation, we measured an increase in ECV_extravascular_ of ∼5% (27 ± 4.1% vs 32 ± 1.9%, p = 0.006, respectively), corresponding to an increase in extravascular lung water volume of ∼40 mL (from 105 ± 19 mL to 143 ± 15 mL, p = 0.048). We found no differences in plasma volume fraction between baseline and mitral regurgitation (43 ± 4.2% vs 46 ± 5.4%, p = 0.26). This nonsignificant 3% increase in plasma volume might reflect distended pulmonary venous volumes in response to the dynamically increased filling pressures. Gadolinium-based ECV, reflecting both the intravascular and extravascular space, was 70 ± 6.7% at baseline and 78 ± 6.9% during mitral regurgitation, p = 0.0026.

**Fig. 6:**
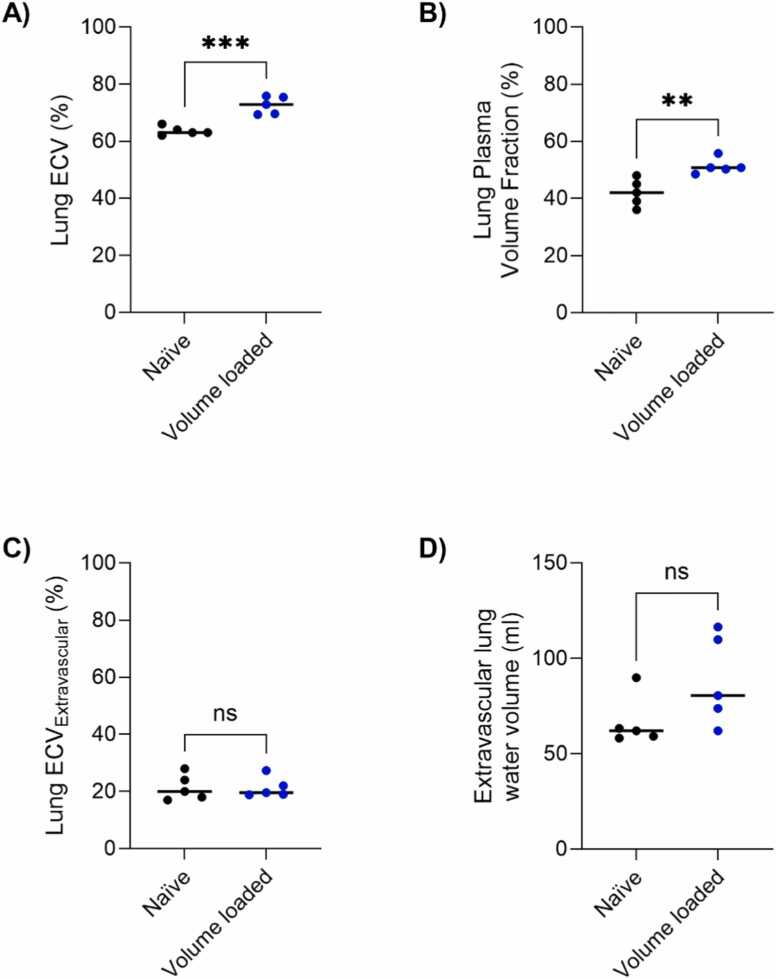
Pulmonary fluid compartments in the naïve vs intravascular volume-loaded porcine model. Lung extracellular volume (ECV) and plasma volume fractions differed when derived using (A) an extracellular gadolinium-based contrast agent and (B) the intravascular contrast agent ferumoxytol, respectively, indicating a higher plasma volume in the pulmonary tissues in the volume-loaded animals. Extravascular (C) ECV and (D) lung water volumes were consistent across the cohorts. Measurements in naïve (shown in black) and volume-loaded (blue) animals were compared using an unpaired t-test: ***p < 0.001, **p < 0.01, *ns* nonsignificant

ECV and plasma volume fraction in naïve vs volume-loaded animals are shown in Fig. 6, disclosing a higher plasma volume fraction in the volume-loaded model (42 ± 4.7% vs 51 ± 2.7%, p = 0.0054), but as expected no differences in ECV_extravascular_ (21 ± 4.6% vs 21 ± 3.6%, p = 0.99) or extravascular lung water (67 ± 13 mL vs 89 ± 24 mL, p = 0.11), which was not induced in these models. Gadolinium-based ECV was 64 ± 1.5% in naïve and 73 ± 3.1% in volume-loaded animals, p = 0.0004.

**Fig. 7:**
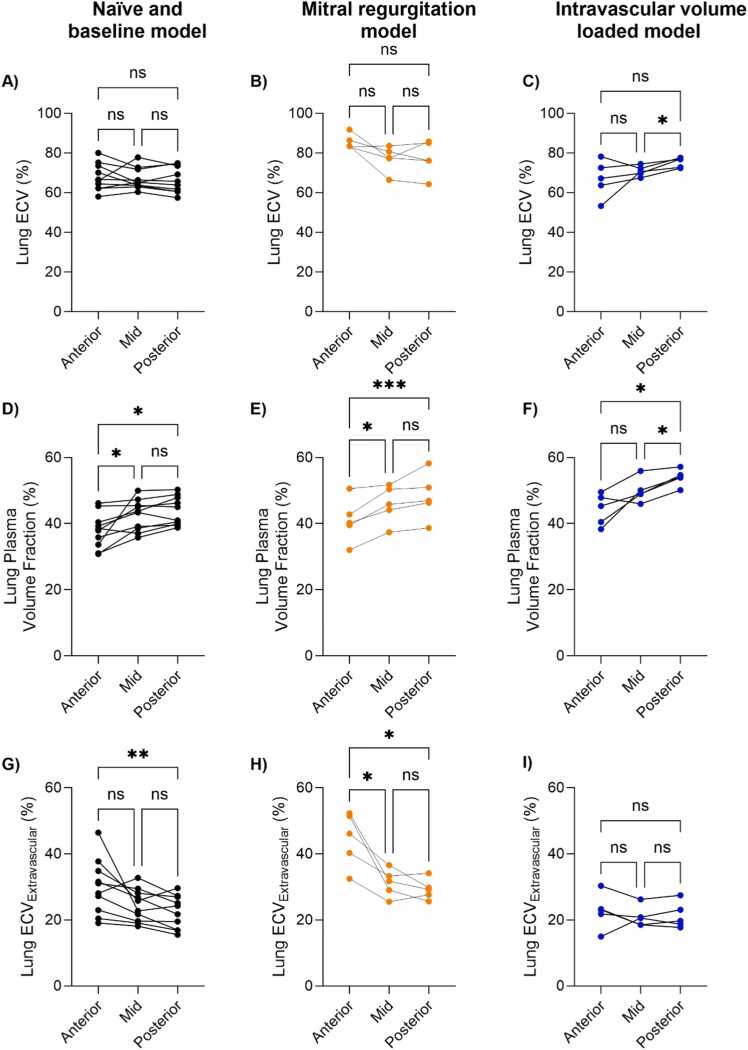
Regional lung extracellular volume (ECV), plasma volume fraction, and extravascular ECV in three anteroposterior sections for the different animal models. Naïve and mitral regurgitation models at baseline are grouped together and shown in black (first column). The mitral regurgitation model animals during valvular opening are shown in orange (middle column), and the intravascular volume-loaded model is shown in blue (third column). One-way ANOVA comparisons: ***p < 0.001, **p < 0.01, *p < 0.05, *ns* nonsignificant, *ANOVA* analysis of variance

The regional lung ECV analysis, shown in Fig. 7 and Table 4, demonstrated a gravitational dependency. The plasma volume fraction was higher posteriorly across all cohorts (naïve or baseline: anterior 38 ± 5.3% vs posterior 44 ± 4.3%; mitral regurgitation: anterior 41 ± 6.7% vs posterior 48 ± 7.1%; volume loaded: anterior 44 ± 4.8% vs posterior 54 ± 2.5%, p < 0.05). On the other hand, ECV_extravascular_ was higher anteriorly in naïve or baseline animals (anterior vs posterior, 30 ± 8.4% vs 22 ± 5.0%, p < 0.001) and during mitral regurgitation (45 ± 8.3% vs 29 ± 3.2%, p = 0.05), but not after volume loading (23 ± 5.5% vs 21 ± 4.0%, p = 0.72). We found differences in mid and posterior lung ECV (71 ± 2.6% vs 75 ± 2.5%, p = 0.02) and plasma volume fractions (50 ± 3.7% vs 54 ± 2.5%, p = 0.01) in the volume-loaded model, but not for any of the other mid vs posterior assessments. In the future, these relationships should be investigated in larger datasets.

**Table 4:** Regional pulmonary fluid quantification.

	Naïve and extravascular lung water model at baseline (n = 10)			Extravascular lung water model during mitral regurgitation (n = 5)			Intravascular volume-loaded model (n = 5)		
	Anterior	Mid	Posterior	Anterior	Mid	Posterior	Anterior	Mid	Posterior
ECV (%)	68±6.8	67±5.4	66±6.4	86±3.8	77±6.5	77±8.6	67±9.4	71±2.6	75±2.5
Plasma volume fraction (%)	38±5.3	43±4.7	44±4.3	41±6.7	46±5.7	48±7.1	44±4.8	50±3.7	54±2.5
ECV_extravascular_ (%)	30±8.4	24±4.9	22±5.0	45±8.3	31±4.2	29±3.2	23±5.5	21±3.2	21±4.0

Consecutive variables are reported as mean ± standard deviation

*ECV* extracellular volume

## 4 Discussion

In this study, we aimed to isolate the pathological extravascular lung water accumulation in heart failure using CMR. We expanded the well-established method of myocardial ECV mapping to the lungs. With a novel dual-contrast agent approach using both an extracellular gadolinium-based contrast agent and the intravascular contrast agent ferumoxytol, we isolate and quantify extravascular ECV and blood plasma volume fractions within the pulmonary tissue. The method’s sensitivity to perturbations in both extravascular and intravascular lung water is demonstrated in porcine models.

### 4.1 Pulmonary fluid quantification

The foundation of our novel dual-contrast method to quantify extravascular lung ECV is the inherently different compartment partitioning of gadolinium-based and ferumoxytol contrast agents, ultimately isolating ECV_extravascular_ through the subtraction of the pulmonary plasma volume fraction from the lung ECV. Another key requirement for ECV quantification is the difference in T1 before and after contrast administration. We, therefore, measured the lung T1 and ΔR1 evolution for each contrast agent and opted for a protocol sequentially acquiring T1 maps at native, gadolinium-based, and ferumoxytol contrast, as this regimen provided clear differentiations in lung T1. As expected, our T1 measurements yielded lower plasma volume fractions compared to lung ECV, reinforcing the different compartment partitioning of these contrast agents.

Upon interactively changing the lung water distribution by inducing mitral regurgitation, we measured expected increases in ECV_extravascular_ and maintained plasma volume fraction. The unchanged plasma volume fraction suggests that the overall pulmonary perfusion was at large maintained during the valvular intervention, but we also note that the increased filling pressures may increase the compliant venous components of the pulmonary circulation. The increase in ECV_extravascular_ was in line with our previous findings using this mitral regurgitation model, where we measured a global increase in lung water using proton density-weighted CMR, which measures both intravascular and extravascular fluid [8]. Our findings of increases in ECV_extravascular_ during mitral regurgitation confirm our then-posed hypothesis, that the global increase in lung water pertained to an increase in extravascular lung water. Although this mitral regurgitation model specifically targeted the accumulation of extravascular fluid in the pulmonary interstitium, we anticipate that this dual-contrast agent method could also detect extravascular fluid in other pulmonary compartments, such as the alveolar or pleural spaces, provided the gadolinium-based contrast agent partitions into those areas. When instead volume loading the intravascular space using a colloid fluid and comparing to naïve controls, we measured differences in plasma volume fractions, but no differences in extravascular ECV. This was also expected, given that no extravascular lung water provocation was performed in these models. Although not detected in the lungs using our dual-contrast agent method, we note that hydroxyethyl starch tissue uptake in a variety of organs, including the lungs, and into intracellular vacuoles has been reported to occur within 30 min of infusion [32].

The gravitational dependency observed on the plasma volume fraction, an indirect reflection of the pulmonary perfusion, aligns with previous findings measuring both lung water and lung perfusion [4], [5], [43], [44], [45], [46]. This pooling of blood along the gravitational axis increases plasma volume fraction and distends vessels in dependent lung regions. With the plasma occupying a larger volume fraction posteriorly, it follows that it occupies a lower volume fraction anteriorly. Concurrently, this leaves space for a larger fraction of ECV_extravascular_, reflecting the pulmonary interstitial fluid, away from gravity-dependent parts of the lungs in animals without intervention (naïve and mitral regurgitation model at baseline). This fluid compartment distribution may therefore not hold in heart failure patients with deranged pulmonary perfusion patterns [44]. In fact, previous findings using positron emission tomography have reported on a range of different extravascular lung water distributions. Schuster et al. have reported both gravity-dependent and U-shaped anteroposterior extravascular lung water distributions in dogs [47], [48], whereas Snashall et al. described a uniform distribution [49]. A combination of regional variations in ventilation, hydrostatic pressure, or osmotic pressures across the lungs may be underlying factors for these variations, but was not assessed in this study. Moreover, we anticipate that distended vessels would not significantly influence extravascular fluid content beyond the pulmonary interstitium, such as in the alveolar or pleural spaces. Notably, fluid in these compartments was not analyzed in the present study.

### 4.2 Interpretation of lung ECV

It is imperative to note that the air volume is not reflected as a volume fraction in the lung ECV, as T1 mapping is insensitive to air. The gadolinium-based lung ECV should therefore not be interpreted as if the lungs are composed of ∼70% fluid, but rather that 70% of the non-air volume is extracellular. Moreover, as ECV is a volume fraction measurement, it is important to consider the potential effect that changes in the total pulmonary water pool could have on the plasma and extravascular fractional calculations. For instance, an increase in total water volume, e.g. due to vascular expansion, could artificially reduce the extravascular volume fraction even if the absolute extravascular volume remains unchanged. Changes in plasma or extravascular ECV should therefore be interpreted in conjunction with the gadolinium-based lung ECV and/or the global lung water volumes derived from proton density-weighted CMR, both of which reflect the combined intravascular and extravascular spaces. Another way to interpret these compartmentalized changes is by multiplying the extravascular volume fraction by the lung water volume, yielding extravascular lung water volume, a direct measure of the total extravascular fluid volume in the lungs.

When calculating extravascular lung water volume, it is furthermore important to mind that proton density-weighted CMR used to measure lung water is sensitive to all fluids, including both the extracellular space and intracellular fluids such as the cytoplasm [4], [5], [8]. Lung water density CMR measures the fluid within a lung segmentation and normalizes signal to surrounding tissue, assuming a 70% musculoskeletal water density [8]. Lung water volume is subsequently calculated by multiplying lung water density with total lung volume from segmentation that includes air and excludes major vasculature. The remaining lung water volume after calculating the extravascular lung water volume compartment should therefore not be interpreted as the pulmonary blood volume, but rather as the sum of the intracellular fluid volume (including both tissue cells and hematocrit) and the plasma volume. Pulmonary blood volume quantification by first-pass perfusion includes all intravascular volume between the ventricles, i.e. both plasma and hematocrit in the lungs, main pulmonary artery, and left atrium [50]. The differences in quantified lung water volume by proton density-weighted CMR and pulmonary blood volumes by first-pass perfusion in this study underscore these inherent technical differences. Future work to further investigate the accuracy of these methods is warranted.

### 4.3 Considerations for clinical application

We anticipate that our method is translatable to humans, and that after careful validation it may be used as a research tool to study the pathophysiological mechanisms behind pulmonary edema. Pulmonary edema is closely related to pulmonary congestion in heart failure at rest and potentially also during exercise stress, and this extravascular lung ECV method may therefore provide pathophysiological insight into exercise-induced dyspnea. Our approach may also be well suited to evaluate treatment efficacy, and we anticipate that intravascular vs extravascular lung water fractions could be repeatedly measured, providing that the gadolinium-based contrast agent and ferumoxytol have been sufficiently cleared. Traces of ferumoxytol can, however, remain in the body for weeks or even months [51], and the potential impact of previous ferumoxytol administration on a repeated dual-contrast ECV assessment remains to be investigated. We also foresee that dual-contrast ECV may provide utility in non-cardiogenic pulmonary edema and other organs, such as in mechanistic studies of myocardial edema.

Carney et al. have previously combined gadolinium-based and ferumoxytol contrast agents in humans to help distinguish colorectal metastases from blood vessels [52]. They removed patients from the scanner for ferumoxytol injection and recognized that this is associated with an additional cost. We acknowledge that the dual-contrast regimen is time-consuming, and patient studies are warranted to establish the potential clinical applicability and added value of extravascular lung ECV. Practical aspects important for clinical applicability include a suitable workflow taking contrast half-life times into account.

Another important aspect of clinical application is the magnetic field strength. Although 0.55T CMR systems are now commercially available, health care settings offering CMR are mostly equipped with 1.5T and 3T scanners. Translating the described method, including the 0.55T-optimized SASHA sequence for pulmonary T1 mapping, might not be applicable for higher field strengths due to a faster T2* signal loss, more off-resonance sensitivity, and increased near-air susceptibility artifacts compared to 0.55T CMR [34]. Whether other lung T1 mapping methods would be feasible, at 0.55T or higher field strengths, remains to be investigated.

## 5 Limitations

This study had several limitations. Sample sizes were small, and we were unable to derive pixel-wise ECV or plasma volume fraction maps due to the workflow of removing animals from the scanner for ferumoxytol infusion. Another limitation is that we did not derive ECV from images covering the entire lungs, but rather approximated this value from three axial slices. This three-slice approach could have implications in describing the average lung ECV and plasma volume fractions, and in turn, the calculation of extravascular lung water volume. Thompson et al. have, however, previously shown that the lung water density measured in one central slice is representative of the global lung water density [3]. Furthermore, our method was validated based on physiological provocations impacting the pulmonary fluid status, but not directly confirmed by independent quantitative measurements. Potential gadolinium-ferumoxytol interactions and how they impact the ECV and plasma volume fraction quantification remain to be investigated. The clinical translation of this method may be limited, as lung T1 mapping might not be feasible at 1.5T and 3T, while 0.55T CMR systems remain relatively scarce.

## 6 Conclusions

In conclusion, extravascular lung ECV can be measured both globally and regionally using a dual-contrast agent CMR-based approach. Extravascular lung ECV changed in the expected directions, consistent with predicted increases in extravascular and intravascular pulmonary fluid after interventions. This measurement may, after further independent validation, comprise a promising method for extravascular lung water volume quantification along with lung water CMR.

## Funding

This study was funded by the 10.13039/100000050National Heart, Lung, and Blood Institute’s (NHLBI) Division of Intramural Research (Z01-HL006257). F.S. received support from NHLBI (K99-HL173575), 10.13039/501100003748Swedish Society for Medical Research, SSMF (PD20-0043), 10.13039/501100003793Swedish Heart-Lung Foundation (2023057224), and Swedish Heart Foundation.

## Author contributions

**Peter Kellman:** Writing – review & editing, Software. **Daniel A. Herzka:** Writing – review & editing, Software, Methodology, Investigation, Data curation, Conceptualization. **Robert J. Lederman:** Writing – review & editing, Supervision, Methodology, Investigation, Funding acquisition, Conceptualization. **Adrienne E. Campbell-Washburn:** Writing – review & editing, Supervision, Project administration, Methodology, Investigation, Funding acquisition, Conceptualization. **Andrea Jaimes:** Writing – review & editing, Methodology, Data curation. **Kendall O’Brien:** Writing – review & editing, Data curation. **Felicia Seemann:** Writing – review & editing, Writing – original draft, Visualization, Validation, Software, Project administration, Methodology, Investigation, Formal analysis, Data curation, Conceptualization. **Rim N. Halaby:** Writing – review & editing, Methodology, Investigation.

## Ethics approval and consent

The study was approved by the Institutional Animal Care and Use Committee at the National Heart, Lung, and Blood Institute, National Institutes of Health.

## Consent for publication

Not applicable.

## Declaration of competing interests

The authors declare the following financial interests/personal relationships which may be considered as potential competing interests: The authors are investigators on a U.S. Government Cooperative Research and Development Agreement (CRADA) with Siemens Healthcare. Siemens participated in the modification of the MRI system from 1.5T to 0.55T. The other authors declare that they have no known competing financial interests or personal relationships that could have appeared to influence the work reported in this paper.

## Data Availability

The datasets used during the current study are available from the corresponding author upon reasonable request.
