# Supplementary material for: Characterizing extravascular lung water—A dual-contrast agent extracellular volume approach by cardiovascular magnetic resonance
Source: J Cardiovasc Magn Reson. 2025 Mar 20;27(1):101883. doi: 10.1016/j.jocmr.2025.101883 (PMC12182819; doi:10.1016/j.jocmr.2025.101883)
Supplement: Supplementary file 1 — Supplementary material [file mmc1.pdf]

Supplemental Material

1. Sequence T1 sensitivity simulations

We performed a Bloch equation simulation of the signal intensity of the saturation recovery singe-shot acquisition (SAHSA) balanced steady state free precession sequence used in this study, with sequence parameters TE/TR/FA 1.18ms/3.0ms/80°, saturation time (TS) array 104x8 – 200x4 – 374x4 ms, FOV 302x393 mm (**Supplemental Figure 1A**). We set T2 = 61 ms, based a previously published average T2 value in the lungs at 0.55T[1].

To assess the variability and robustness of the SASHA T1 mapping, we performed a Monte Carlo simulation based on the magnetizations for six different T1 values (200, 400, 600, 800, 1000, and 1200 ms). The Bloch equation simulated data at were fit to the 3-parameter model $S0=A-B\cdot e^{(-TS/T1)}$, using non-linear least squares optimization. An estimate for parameters A and B was obtained from the fit, and these parameters were then used to generate synthetic data for 10.000 Monte Carlo simulations per T1 value. Gaussian noise with was added to the signal, based on the simulated signal divided by the apparent SNR (mean 73), which was measured from the experimental data. For each iteration, T1 was re-estimated by fitting the noisy data to the model. The standard deviation of the resulting distribution of T1 estimates was calculated (**Supplemental Figure 1B**). The average standard deviation as a percent of the simulated T1 values was 6.7±1.2%, indicating that the sequence is sensitive to a range of physiological T1 values pre and post contrast at 0.55T.

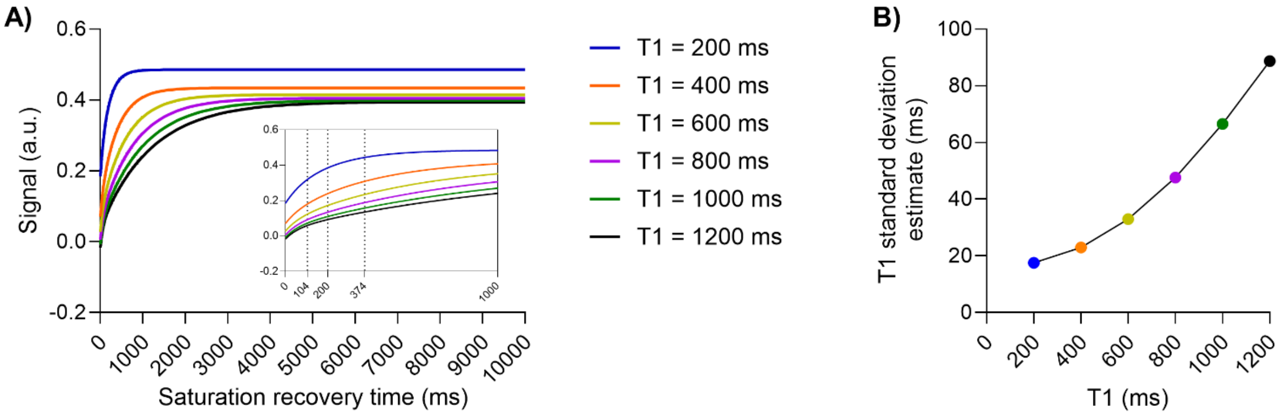

Supplemental Figure 1. A) Bloch equation simulation of the SASHA signal intensities, demonstrating the theoretical signal intensities across the range of T1 values measured in the lungs and blood in this study. Insert shows the simulation with vertical dotted lines at the saturation recovery times from the end of the saturation pulse to the center of k-space. **B)** The Monte Carlo simulated standard deviations for a range of T1 values.

2. Derivation of extracellular volume fraction equation

The extracellular volume fraction (ECV) of a given tissue can be derived through the blood-tissue partition coefficient (λ), defined as the ratio[2]:

$$\lambda = \frac{C_{tissue}}{C_{blood}}, \qquad (Eq. 1)$$

where C is the extracellular contrast agent concentration. The tissue T1 relaxation (R1 =1/T1) after contrast administration can be described as a function of the native R1, i.e. R1 before contrast administration, the contrast compound relaxivity r_1, and C as[3]:

$$R1_{contrast} = R1_{native} + r_1 \cdot C \qquad (Eq. 2)$$

Hence, the blood-tissue partition coefficient can be expressed as

$$\lambda = \frac{(R1_{contrast} - R1_{native})_{tissue}}{(R1_{contrast} - R1_{native})_{blood}} = \frac{\Delta R1_{tissue}}{\Delta R1_{blood}}. \qquad (Eq. 3)$$

Moreover, the tissue contrast concentration can be described as a weighted sum of the *k* compartments multiplied by their respective volume fractions ($V_{\%}$)[4]:

$$C_{tissue} = \sum_{n=1}^{k} C_{compartment,n} \cdot V_{\%compartment,n}. \qquad (Eq. 4)$$

This equation can also be expressed as

$$C_{tissue} = \frac{\Delta R1_{tissue}}{r_1} = \sum_{n=1}^{k} \frac{\Delta R1_{compartment,n}}{r_1} \cdot V_{\%compartment,n}, \qquad (Eq. 5)$$

From which it follows that

$$C_{tissue} = \Delta R1_{tissue} = \sum_{n=1}^{k} \Delta R1_{compartment,n} \cdot V_{\%compartment,n} \qquad (Eq. 6)$$

For blood, we have a 2-compartment model consisting of plasma and red blood cells (RBC):

$$C_{blood} = C_{plasma} \cdot V_{\%plasma} + C_{RBC} \cdot V_{\%RBC}. \qquad (Eq. 7)$$

Since we are working with extracellular contrast agents, no contrast is present in the intracellular space, meaning in this case we have C_{RBC}=0. From a blood draw we can measure the hematocrit (hct), which correspond to the volumetric fraction of packed red blood cells, i.e. $V_{\%RBC} = hct$. Hence, it follows that

$$V_{\%plasma} = 1 - V_{\%blood} = 1 - hct \qquad (Eq. 8)$$

and

$$C_{plasma} = \frac{C_{blood}}{1 - hct}. \qquad (Eq. 9)$$

Assuming contrast equilibrium in the studied tissue, i.e. equal contrast concentration in the intravascular and extravascular spaces ($C_{plasma} = C_{extracellular,extravascular}$) and that $V_{\%plasma} \ll V_{\%extracellular,extravascular}$, we have:

$$\lambda = \frac{C_{tissue}}{C_{blood}} = \frac{C_{plasma} \cdot V_{\%plasma} + C_{extracellular,extravascular} \cdot V_{\%extracellular,extravascular}}{C_{plasma} \cdot (1 - hct)} =$$

$$= \frac{V_{\%plasma} + V_{\%extracellular,extravascular}}{(1 - hct)}. \qquad (Eq. 10)$$

The entire tissue ECV is composed of the plasma and the extracellular extravascular spaces, ultimately yielding the well-established expression

$$\mathrm{ECV} = V_{\%plasma} + V_{\%extracellular,extravascular} =$$

$$= \lambda \cdot (1 - \mathrm{hct}) = (1 - hct) \cdot \frac{\Delta R1_{tissue}}{\Delta R1_{blood}}. \qquad (Eq. 11)$$

2.1 Ferumoxytol-based ECV in the blood pool

Ferumoxytol ("Fex") is an extracellular contrast agent that remains within the intravascular space, meaning its partitioning is limited to the blood plasma. With $C_{RBC} = \frac{\Delta R1_{RBC}}{r_1} = 0$ and $V_{\%plasma} = 1 - hct$, per equation 6 the 2-compartment model of the intravascular space

$$\Delta R1_{blood\,pool} = \Delta R1_{plasma} \cdot V_{\%plasma} + \underbrace{\Delta R1_{RBC} \cdot V_{\%RBC}}_{=0}. \qquad (Eq. 12)$$

Ultimately, we can express the change in blood pool R1 following ferumoxytol administration as

$$\Delta R1_{plasma,Fex} = \frac{\Delta R1_{blood\,pool,Fex}}{1 - hct}. \qquad (Eq. 13)$$

2.2 Ferumoxytol-based ECV in the lung tissue

For the purposes of the lungs, we consider a 4-compartment model, divided into the intravascular (i.e. plasma and red blood cells) and extravascular (i.e. extracellular and intracellular lung parenchymal spaces) spaces. Note that the air compartment is not considered in this model, as air does not provide any CMR signal. Again, since ferumoxytol ("Fex") is an extracellular intravascular contrast agent, its partitioning is limited to the blood plasma, we have

$$\Delta R1_{lung\ tissue,Fex} = \Delta R1_{plasma} \cdot V_{\%plasma,tissue} + \underbrace{\Delta R1_{RBC} \cdot V_{\%RBC,tissue}}_{=0} + \\ + \underbrace{\Delta R1_{extracellular,extravascular} \cdot V_{\%extracellular,extravascular}}_{=0} + \\ + \underbrace{\Delta R1_{intracellular,extravascular} \cdot V_{\%intracellular,,extravascular}}_{=0} = \Delta R1_{plasma} \cdot V_{\%plasma,tissue} \\ (Eq.\ 14)$$

Note that $V_{\%plasma,tissue}$ is not (1-hct), as it reflects the plasma volume fraction across a the entire tissue sample, not just within the blood pool. Using equation 12, the plasma volume fraction, $V_{\%plasma,tissue}$, can be described as

$$Plasma\ volume\ fraction = V_{\%plasma,tissue} = \frac{\Delta R1_{lung\ tissue,Fex}}{\Delta R1_{plasma,Fex}} = \\ = (1 - hct) \cdot \frac{\Delta R1_{lung\ tissue,Fex}}{\Delta R1_{blood\ pool,Fex}}. \qquad (Eq. 15)$$

2.3 Gadolinium-based ECV in the lung tissue

Gadolinium-based contrast agents ("Gad") partition in the entire extracellular space, i.e. both in the extravascular and intravascular space. For the lungs, equation 6 can therefore be expressed as

$$\Delta R1_{lung\ tissue,Gad} = \Delta R1_{plasma} \cdot V_{\%plasma,tissue} + \underbrace{\Delta R1_{RBC} \cdot V_{\%RBC,tissue}}_{=0} + \\ + \Delta R1_{extracellular,extravascular} \cdot V_{\%extracellular,extravascular} + \\ + \underbrace{\Delta R1_{intracellular,extravascular} \cdot V_{\%intracellular,extravascular}}_{=0} = \Delta R1_{plasma} \cdot V_{\%plasma,tissue} + \\ + \Delta R1_{extracellular,extravascular} \cdot V_{\%extracellular,extravascular}. \qquad (Eq. 16)$$

Considering equation 10, we can formulate the blood-tissue partition coefficient as

$$\lambda = \frac{\Delta R1_{lung\ tissue,Gad}}{\Delta R1_{blood\ pool,Gad}} = \frac{V_{\%plasma,tissue} + V_{\%extracellular,extravascular}}{(1 - hct)}. \qquad (Eq.\ 17)$$

2.4 Extravascular ECV in the lung tissue

Rearranging equation 17 yields

$$V_{\%extracellular,extravascular} = (1 - hct) \cdot \frac{\Delta R1_{lung\ tissue,Gad}}{\Delta R1_{blood\ pool,Gad}} - V_{\%plasma,tissue}, \qquad (Eq.\ 18)$$

which combined with equation 15 results in the following equation describing the pulmonary extravascular ECV:

$$V_{\%extracellular,extravascular} = (1 - hct) \cdot \left(\frac{\Delta R1_{lung\ tissue,Gad}}{\Delta R1_{blood\ pool,Gad}} - \frac{\Delta R1_{lung\ tissue,Fex}}{\Delta R1_{blood\ pool,Fex}}\right) \quad (Eq.\ 19)$$

or

$$ECV_{extravascular} = ECV - Plasma\ volume\ fraction. \qquad (Eq.\ 20)$$

3. Supplemental References

1. Campbell-Washburn AE., Ramasawmy R., Restivo MC., et al. Opportunities in interventional and diagnostic imaging by using high-performance low-field-strength MRI. Radiology 2019;293(2):384–93. Doi: 10.1148/radiol.2019190452.

2. Arheden H., Saeed M., Higgins CB., et al. Measurement of the Distribution Volume of Gadopentetate Dimeglumine at Echo-planar MR Imaging to Quantify Myocardial Infarction: Comparison with 99m Tc-DTPA Autoradiography in Rats. Radiology 1999;211(3):698–708. Doi: 10.1148/radiology.211.3.r99jn41698.

3. Flett AS., Hayward MP., Ashworth MT., et al. Equilibrium Contrast Cardiovascular Magnetic Resonance for the Measurement of Diffuse Myocardial Fibrosis. Circulation 2010;122(2):138–44. Doi: 10.1161/CIRCULATIONAHA.109.930636.

4. Jerosch-Herold M., Sheridan DC., Kushner JD., et al. Cardiac magnetic resonance imaging of myocardial contrast uptake and blood flow in patients affected with idiopathic or familial dilated cardiomyopathy. Am J Physiol Heart Circ Physiol 2008;295(3):H1234–42. Doi: 10.1152/ajpheart.00429.2008.
